# Supplementary material for: Binucleated human hepatocytes arise through late cytokinetic regression during endomitosis M phase
Source: J Cell Biol. 2024 May 10;223(8):e202403020. doi: 10.1083/jcb.202403020 (PMC11090133; doi:10.1083/jcb.202403020)
Supplement: Table S1 — shows organoid culturing media. [file JCB_202403020_TableS1.docx]

**Table S1. Organoid culturing media**

|  | **Component** | **Catalog number** | **Supplier** | **Concentration** |
| --- | --- | --- | --- | --- |
| Chol-Org  Expansion Medium | Advanced DMEM/F12 | 12634 | Gibco |  |
|  | HEPES | 15630 | Gibco | 10 mM |
|  | GlutaMAX | 35050 | Gibco | 1x |
|  | Penicillin/Streptomycin | 15140 | Gibco | 100 U/mL |
|  | RSPO1 conditioned medium | - | (Made in house) | 10% |
|  | B27 minus vitamin A | 12587-010 | Thermo Scientific | 1x |
|  | Nicotinamide | 72340 | Sigma Aldrich | 10 mM |
|  | N-acetylcysteine | A7250 | Sigma Aldrich | 1.25 mM |
|  | FGF10 | 100-26 | Peprotech | 100 ng/mL |
|  | A83-01 | 2939 | Tocris | 5 µM |
|  | FSK | 1099 | Tocris | 10 µM |
|  | HGF | 100-39 | Peprotech | 25 ng/mL |
|  | EGF | AF-100-15 | Peprotech | 50 ng/µL |
|  | Gastrin I | 3006 | Tocris | 10 nM |
| Hep-Org  Expansion Medium | Advanced DMEM/F12 | 12634 | Gibco |  |
|  | HEPES | 15630 | Gibco | 10 mM |
|  | GlutaMAX | 35050 | Gibco | 1x |
|  | Penicillin/Streptomycin | 15140 | Gibco | 100 U/mL |
|  | RSPO1 conditioned medium | - | (Made in house) | 15% |
|  | B27 minus vitamin A | 12587-010 | Thermo Scientific | 1x |
|  | Nicotinamide | 72340 | Sigma Aldrich | 10 mM |
|  | N-acetylcysteine | A7250 | Sigma Aldrich | 1.25 mM |
|  | Y-27632 | Y0503 | Sigma Aldrich | 10 µM |
|  | CHIR99021 | 4423 | Tocris | 3 µM |
|  | HGF | 100-39 | Peprotech | 50 ng/mL |
|  | FGF7 | 100-19 | Peprotech | 100 ng/mL |
|  | FGF10 | 100-26 | Peprotech | 100 ng/mL |
|  | A83-01 | 2939 | Tocris | 2 µM |
|  | EGF | AF-100-15 | Peprotech | 50 ng/mL |
|  | TGF-α | 100-16A | Peprotech | 20 ng/mL |
|  | Gastrin I | 3006 | Tocris | 10 nM |
