# Supplementary material for: Binucleated human hepatocytes arise through late cytokinetic regression during endomitosis M phase
Source: J Cell Biol. 2024 May 10;223(8):e202403020. doi: 10.1083/jcb.202403020 (PMC11090133; doi:10.1083/jcb.202403020)
Supplement: Table S2 — shows oligo sequences of gRNAs, primers used for genotyping, and primers used for qPCRs. [file JCB_202403020_TableS2.docx]

**Table S2. Oligo sequences**.

| **Name** | **Sequence** | **Reference** |
| --- | --- | --- |
| gRNA for TUBB CRISPaint | CACCGAGGCGGCGAGGACGACTA | Artegiani et al., 2020 |
| gRNA for CDH1 CRISPaint | CACCGAGGCGGCGAGGACGACTAG | Artegiani et al., 2020 |
| gRNA for E2F7 CRISPR base editing | AAGAATCAGTATGGCTGGCA | This paper |
| gRNA for E2F8 CRISPR base editing | AAAGTACAGCTGGCAAGATC | This paper |
| Primer E2F7 genotyping FWD | AGGCCACCAGTTTCAGCTGCTG | This paper |
| Primer E2F7 genotyping REV | AGTGGGCAGTCCAGTTGGGGAG | This paper |
| Primer E2F8 genotyping FWD | TCTGCGCCCAGTAGCCCTATCA | This paper |
| Primer E2F8 genotyping REV | ATGAGGGACGGTGCTGTCAGCT | This paper |
| Primer E2F7 qPCR FWD | CAGGCAGCCCAGACTAGATTT | This paper |
| Primer E2F7 qPCR REV | GTTCCGCTTGCTGTCTGTCA | This paper |
| Primer E2F8 qPCR FWD | AGGCCAAAGACTGTATACACGAACA | This paper |
| Primer E2F8 qPCR REV | TCGTAAATGCGTCGACGTTCAACA | This paper |
